# Carbohydrate Starvation in Typhoid Fever

**Published:** 1896-04-04

**Authors:** Eric Pritchard


### CARBOHYDRATE STARVATION IN TYPHOID FEVER.

By ERIC PRITCHARD, M.A., M.B., B.Ch.(Oxon.).

(*Continued from page 342.*)

SUCH fermentation changes are particularly liable to occur when starch in any form is given as a food, and, to a certain extent, when cane or grape-sugar takes its place. But with the natural sugar of milk, or lactose, fermentative changes are slow to appear; and on this ground there is no reason why the deficiency of carbohydrate in the food should not be made good by this means. For these reasons it has been my practice for some time past to bring up the total quantity of the carbohydrate food to nearly that which is required in conditions of health by the addition of a corresponding quantity of lactose to the milk. I have myself adopted this practice in fifteen cases of typhoid, and on my suggestion I have seen it employed in several other cases. The results have been uniformly good; tympanitis from fermentation rarely appeared, although in a few of the cases towards the end tympanitis made its appearance probably from nerve exhaustion. The advantage of this method was, however, in all cases clearly manifest during the convalescent stage; there was a conspicuous absence of that craving for solid food, which is usually both a danger to the patient and a source of worry to the physician. Although it is difficult to compare the condition of nutrition of patients treated on the two plans, namely, with carbohydrate starvation and without, I think one may say with certainty that it is not to the disadvantage of the latter. The employment of lactose has further advantages if from improvement of the powers of digestion it becomes necessary to peptonise the food; because the subsequent addition of lactose makes it undoubtedly more palatable to the patient.

One's aim, therefore, should be to keep the circulating reserve of carbohydrate as high as possible, so as to protect the tissue cells from unnecessary destruction, a destruction which in all fever conditions is unduly great. Is there any probability of overdoing this stocking of the blood with carbohydrate? Can too much be absorbed from the intestinal tract, and would such an event in itself intensify the fever or introduce other complications? All these questions can probably be answered in the negative, for any "luxus" absorption of such food-stuffs can only result in the storage of glycogen or fat, or in immediate oxidation, neither of which events is calculated to influence the course of the fever. In typhoid fever, however, the powers of absorption, even of so soluble a substance as sugar, are comparatively feeble in the intestine, and consequently, however much of the carbohydrate be introduced into the stomach, it is more than improbable that an excessive amount should be absorbed. But, if it be not absorbed, an easily fermentable substance of this kind in the intestine is a serious source of danger, since it may lead to tympanitis and other complications. Hence the object of the physician is to supply to the stomach as much as it, together with the intestine, can absorb, without leaving any excess to undergo lactic or other fermentation. The practical difficulty is, of course, to discover the exact quantity suitable for each particular case. No arbitrary rule can be formulated, but in each case the physician must exercise his own discretion. Tympanitis in the early stages of the fever is a certain guide of fermentation in the intestine, and the fermentation is almost sure to be of carbohydrate origin. If this cannot be controlled by the exhibition of intestinal antiseptics, such as salol, carbolic acid, calomel, &c., the total quantity of carbohydrates must be reduced. Lactose may, of course, undergo lactic acid fermentation, but it is so readily converted into galactose, and absorbed into the system, that fermentation is far less likely to occur with this carbohydrate than with any other that can be advantageously employed. The mere boiling of lactose is sufficient to invert it without the addition of the ferment. The addition of 1 per cent. hydrochloric acid before boiling accelerates the process, and, although I have not tried this method myself, it is worth considering whether this plan should not be adopted in all cases in which sugar of milk is added to the diet. In the form of galactose it can be immediately absorbed from the mouth, the stomach, or intestines, and only under exceptional circumstances can it undergo the lactic acid or other fermentative change.

Now as to the question of the amount of sugar which it is desirable to add to the milk. My own practice has been to dissolve one ounce of lactose in boiling water, and to add this to one pint of lightly skimmed milk, and to give during the twenty-hour hours five pints of this modified milk. By this means the patient obtains about double as much carbohydrate food as he would were he merely to take two and a half pints of simple milk; the fat is decreased by the skimming to proportions such as can be assimilated by the system, and the total quantity of proteid is the same as that usually given in exclusive milk diet. If the patient complains of much thirst there is no reason why the milk should not be further diluted. A free flushing of the system with water is the best means of removing the impurities from the blood. Should tympanitis supervene the quantity of milk must be reduced. If undigested curd of milk is noticed in the stools whey should be substituted for milk, and the proteid deficiency made good by meat juice or other soluble albumen. If this alteration does not meet the case, the milk should be peptonised.

To sum up. Milk, whether peptonised or plain, is the most convenient food for typhoid patients. Ordinary milk contains too much fat, and should therefore be skimmed: it does not contain sufficient carbohydrate, and should therefore receive a supplemental quantity of sugar of milk—one ounce to the pint. The total quantity of milk in the day should be not less than two and a half pints, and the total quantity of fluid not less than five pints. The milk should, therefore, be diluted with an equal quantity of water.

